# The Universality of Lying

**Published:** 1880-10

**Authors:** Ben. H. Pratt


### THE BISTOURY.

Vol. XVI. ELMIRA, N. Y., OCTOBER, 1880. No. 3.

### Contributions.

### THE UNIVERSALITY OF LYING.

BEN. H. PRATT.

The quickest way to make a man mad is to call him a liar. If I walk up to a man and say "you are a liar!" he'll knock me over. If an editor intimates in his columns that one of his townsmen is a liar, ten to one that editor will suffer for it, physically or otherwise. People have a singular aversion to be esteemed liars by their fellow men. It is common to consider lying as one of the meanest traits a man can possess, and a liar as the meanest man in the community.

Considering the universality of lying, and its necessity in the economy of life, the unpopularity of the habit is something unaccountable. I should not call it a habit, however, for it is more than that; it is a characteristic of the human race the world over. All classes and con ditions of men lie in a more or less degree, and when lying makes mankind happier, and is encouraged on every hand, I am at a loss to know why it should be, theoretically, so unpopular; while truthfulness, which is so exceedingly rare, and productive of so much misery, should be regarded as typical of a high order of manhood and womanhood.

Lying, as society exists to-day, is essential to the happiness of mankind. We could not maintain any thing like civilization without it. The world would become all awry, and the dwellers therein would have each other by the ears in less than a fortnight, if lying were abolished for that period. Families would be broken up. Children would rise up against their parents, and parents against their children. Husbands could not tolerate wives, nor wives husbands. Brothers and sisters, cousins and aunts, nephews and nieces, relatives of every sort would be driven to annihilate each other. There could be no loves, nor friendships, nor intimacies of any kind whatever between members of the human family, did we not tacitly recognize the fact that to a certain extent we must lie in order to endure each other. If the whole world should speak nothing but absolute truth for two weeks, the whole fabric of our social, business and political economy would fall to the ground, and association would become an impossibility. Governments, commerce, society, friendships, attachments, admirations, loves, are founded upon and sustained by falsehood. It is impossible to conceive how man, constituted as he is, could live at peace with his fellow man without lying. The decalogue recognizes this impossibility, and hence we do not find "Thou shalt not lie," but "Thou shalt not bear false witness," a very wise modification. It says "Thou shalt not steal"—"Thou shalt not have any other gods"—"Thou shalt not commit adultery," &c.—but no such direct forbiddance of lying.

All men, women and children lie. It is natural for them to lie. They cannot help lying. No one with perspicacity enough to perceive the consequences of absolute truthfulness at all times and on all occasions would help lying if he could. It's all very well to say that while some lies are sinful, others are not; that there is a broad distinction between wilful lying and lies which we don't mean to tell, and which are so innocent in their results that they are not regarded as heinous, and all that sort of thing. The distinction between a malicious lie—one intended to do one's neighbor harm—and those lies which we call fibs, prevarications, innocent deceptions, and all that—is very fine, and may ease the consciences of many; but where will you draw the line between lies that may, and lies that may not, be uttered. Each of us will draw his own line, and I think it far better and far manlier to admit that his lies are essential to human happiness, and stop there—call all perversions of the truth, lies, and get along with them the best we can. It won't do to sweepingly assert that it is wicked to lie when lying is of so much benefit to ourselves and our neighbor; nor will it do to say that it is right to lie, when lying may work such terrible harm among our associates. I confess I cannot assent either, nor can I deny either. I recognize the fact that we all lie and must keep on lying, but I do not pretend to say that it is right or wrong, and I cannot see where anyone can draw the moral line between the lies that border upon the harmful and those that border on the innocent, and direct us just how much we may and how much we may not lie. Still, harsh as the saying may seem, there's no question whatever but what we all lie, sometimes unnecessarily, often necessarily, and more than this, we approve of others lying and encourage them in it.

Children lie before they can talk. They act out their little lies in the cradle. About the first intelligent thing a child does is to lie. Didn't you ever watch a baby's way of telling a whopper? This is one way of doing it. Suppose the baby has just been fed, and is laid away for a few moments of maternal rest. Left alone, the child squalls. It's a peculiar squall, the kind it makes when hungry. Every mother recognizes it at once as distinct from the mad squall, or the sick squall, or the impatient squall, or the squall of pain, or any of the fifty distinct kinds of squally which go to make up a baby's vocabulary of mandatory vociferation. It wants to be picked up, but knowing that a squall of impatience or of anger will not touch the mother's sympathy, it gives the hungry squall, notwithstanding its stomach is already fuller than it ought to be. The hunger cry is electric in its effect upon a mother. Mothers can stand anything better than the intimation that their babes are hungry. Stuffing babies to repletion is a maternal weakness that will never be overcome by the human mother. It's sad to know this, on the baby's account, but it's so. This hunger cry is a baby's acted lie, as is proved in two minutes after the child is taken up and offered food. It won't take a drop, but will dally with spoon or other medium of receiving food, and sputter and coo its satisfaction over the result of its lie, just as plainly as a clothier, a doctor, an editor or a lawyer that has accomplished a successful lie and gotten the price of it. Note its twinkling eye, its twitchy lip, its whole air of exultation over the fact that it has lied and reaped the tangible fruits thereof. Babies lie in this way and other ways to secure their objects, and as they grow older and wiser, their lies grow in ingenuity and wonderfulness until children become as accomplished liars as grown people generally are. And yet we pretend to be astonished when we catch a boy or girl in a square, downright lie, and we act as though this were the first lie we ever heard. And the child is as really astonishished as we pretend to be, when we tell it that good people—our kind, of course—never lie. It's natural for a child to lie. It inherits lying, and is taught lying every day of its existence by its own parents, relatives and associates. We read about absolutely truthful children, but we don't come across them in life. Does it never occur to those who are so scrupulous about having their children always tell the downright truth, that they are continually teaching them to lie? What is a lie? That which is not true, or conveys to another an impression contrary to fact. Now, how often does your child catch you telling an absolute falsehood without your even being aware that you have told one. Deception of any sort is lying. It is an effort to misrepresent facts. You train your children to behave better in company, or when visiting, than they behave at home. By so doing you teach them to impress upon others that they are better behaved than they really are. A mother will put half a peck of false hair on her head in the presence of a child, and the child knows her mother does it to make people believe it's her own live hair. You send Bridget to the door to tell a caller that you are not at home. You tell your daughter not to play with those nasty little Smith brats, and you talk to Mrs. Smith, in the presence of your daughter, about her dear little girls. You talk about that coarse and dowdy Mrs. Brown, but you kiss her and say you are so glad to see her when she comes to call. You must lie to those who come to see you and to those whom you go to see. You daren't tell the truth, and if you did you'd rue it for all time. You must appear to like what you dislike, and vice versa, or the social circle would become a howling bedlam. You must pretend to love whom you hate, and you teach your children to do so, and yet you make a terrible ado if once you catch your child in a lie.

Suppose your children should, by some miraculous or preternatural process, grow up the truthful beings you imagine they should be. They would mortify you to distraction and worry you to death. If your children told their playmates just what they think of them, and told your friends whom they meet just what they know you think of them, and had heard you say about them, how long could you live in your present neighborhood. Suppose your children were absolutely truthful and made candid remarks concerning those who visit you, and in their presence, how long would you have a visiting acquaintance in the community? Suppose Johnny should come from the kitchen while you and your company are at tea and tell you that Bridget dropped the platter of oysters into the coal scuttle, but had picked them out and they are now all right? Suppose your truthful daughter would follow this up with the inquiry as to why you don't take out your teeth when you eat, just as you do when you haven't company! It wouldn't do at all. Truth would destroy the whole social fabric. The fact is, you have to lie to your best friends and tell the truth about and to your enemies. Men may tell the truth about other men's business, but in their own business it is the most inconvenient and unfinancial habit men can have. It is rather expected of men that they be not reliable in speaking of their business, and we have heard professing christians assert that they didn't consider business lies as the kind we are forbidden to use.

Every day we are absolutely obliged, in order to keep peace in the community, to talk and act toward those we meet as if we believed there wasn't an imperfection of the most trivial sort in their composition, mental, moral or physical. You know we are. And we have to lie terribly to do it. There's no escape from it. Society would go to the dogs in a trice if we did not lie at least nine tenths of the time. No one can speak his real mind, though many pretend to do so, bragging that they are plain people who tell the truth regardless of consequences; blunt people who say just what they think. But they never lie so outrageously as when they talk this way.

It's a sad state of things, looked at from the absolutely moral point of view, but what can we do? Tell the truth and be miserable from youth to the grave? Never lie, and suffer the bitterest persecutions of our kind? Would it be policy for any community of people to agree to tell the truth at all times? I think that community would become extinct through assassinations in the course of three months. I have no remedy to propose, but would be glad to hear some one suggest what we who hate to lie, but are obliged to, can do under the distressing nature of the circumstances.

